# Digital Phenotyping of High-Risk Gaming Behavior Using Wearable Devices

**DOI:** 10.31083/AP48256

**Published:** 2026-06-30

**Authors:** Yijie Song, Yawen Shi, Yiqun Tu, Haidi Shan, Chuanning Huang, Yue Wang, Shuo Li, Zhekai Li, Hang Su, Na Zhong, Tianzhen Chen, Min Zhao, Jiang Du

**Affiliations:** ^1^Shanghai Mental Health Center, Shanghai Jiao Tong University School of Medicine, 200030 Shanghai, China; ^2^Shanghai Changning Mental Health Center, Affiliated Mental Health Center of East China Normal University, 200335 Shanghai, China; ^3^Shanghai Key Laboratory of Psychotic Disorders, 200030 Shanghai, China; ^4^CAS Center for Excellence in Brain Science and Intelligence Technology (CEBSIT), Chinese Academy of Sciences, 200031 Shanghai, China

**Keywords:** internet gaming disorder, wearable electronic devices, machine learning, digital health

## Abstract

**Background:**

Gaming addiction is a serious mental health issue. Early identification of high-risk gaming behaviors (HRGB) is crucial for prevention, yet research using wearable data for detection remains limited. This study analyzed the temporal dynamics of gaming behavior and health indicators using 28-day summary statistics derived from longitudinal data in young adults with HRGB, thereby providing a basis for gaming disorder prevention and intervention.

**Methods:**

This 28-day longitudinal study employed a self-designed system implemented on the Huawei Research Platform for participant recruitment and data collection. Participants completed daily mobile app questionnaires between 18:00 and 23:00 assessing gaming time, mood, and sleep, while a smart band continuously collected physiological and activity data. Emotional states were assessed weekly using the Patient Health Questionnaire-9 (PHQ-9) and the Generalized Anxiety Disorder-7. Machine learning models were applied to compare the predictive performance of active (self-report), passive (wearable), and combined data in identifying HRGB, and to determine key predictive factors relevant for intervention.

**Results:**

The final sample comprised 37 participants in the HRGB group and 23 in the non-HRGB group. Significant between-group differences were observed across multiple indicators. Combined data yielded the highest area under the receiver operating characteristic curve (AUROC) in five of six models (logistic regression: 0.86 vs 0.81). Wearable-derived data enhanced early classification of HRGB, with depression severity (PHQ-9), heart rate, and sleep metrics emerging as potential markers. A one-standard-deviation increase in PHQ-9 scores was associated with a 3.51-point increase in Gaming Disorder Screening Scale (GDSS) scores (89% credible interval [CrI], 1.85–5.20), while a higher heart rate index was associated with a 1.36-point increase in GDSS scores (89% CrI, 0.13–2.60). The population attributable fraction was 15.51% for PHQ-9.

**Conclusions:**

Wearable data enhances early HRGB classification. PHQ-9, heart rate, and sleep metrics are key predictors, showing clinical utility.

## Main Points

1. Multimodal fusion of wearable-derived physiological and self-reported psychobehavioral data boosts the binary classification performance of the machine learning model, validating digital phenotyping for early detection of high-risk gaming behaviors (HRGB).

2. Patient Health Questionnaire-9 (PHQ-9), heart rate index, and deep sleep proportion were the strongest predictors among all collected variables, with each standard deviation increase in PHQ-9 score associated with a 3.51-point rise in the Gaming Disorder Screening Scale score.

3. Alleviating depressive symptoms in high-risk individuals could prevent approximately 15.5% of HRGB cases, suggesting that depression intervention is a potential key prevention strategy.

4. Elevated heart rate and reduced deep sleep proportion in HRGB may suggest heightened sympathetic activity and disrupted sleep architecture, which could implicate autonomic nervous dysregulation in gaming behavior mechanisms.

## 1. Introduction

Gaming addiction has increasingly been recognized as a major public health challenge, particularly among adolescents and young adults [1,2]. Problematic gaming behaviors are rising due to the widespread availability of digital devices and immersive games, resulting in psychological impairment, social disruption, and poor academic or occupational performance [3,4]. The World Health Organization’s inclusion of “gaming disorder” in the International Classification of Diseases underscores the seriousness of this condition [5,6]. Early identification of high-risk gaming behaviors (HRGB) is clinically critical because it provides a window for intervention before patterns become entrenched. Detecting subclinical vulnerability enables targeted prevention, such as psychoeducation and cognitive-behavioral techniques, to mitigate the progression to a full-blown disorder [7]. Despite these compelling reasons, current screening methods often rely on self-reported retrospective data, which are susceptible to bias and underreporting [8,9]. Developing objective, continuous, and ecologically valid assessment tools is critical for capturing dynamic real-world data, thereby revolutionizing early detection and supporting scalable public health interventions.

**Fig. 1.**
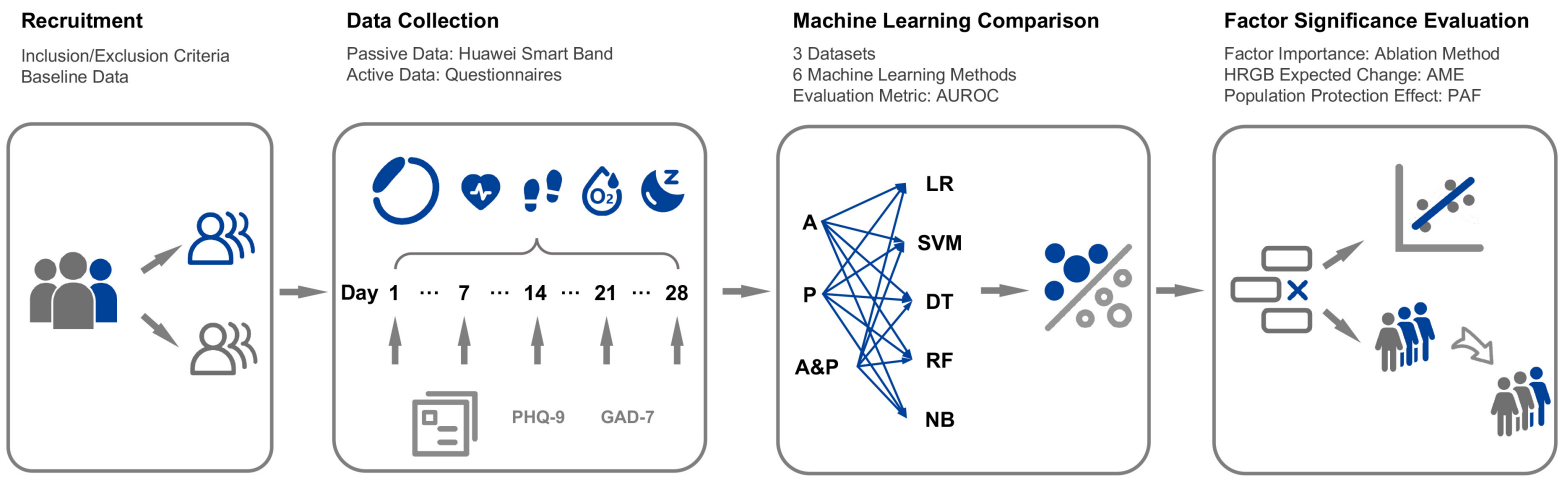
**Study design and data analysis flow**. Recruitment: HRGB and non-HRGB controls are recruited according to predefined inclusion/exclusion criteria, and baseline data are collected at the same time. Data Collection: Game behavior-related physiological indicators such as heart rate, daily steps, blood oxygen saturation, and sleep metrics are passively collected via Huawei Smart Band. Emotion and stress scores are actively collected through weekly questionnaires. Machine Learning Comparison: three datasets (active, passive, and combined) are used to evaluate the classification performance of six machine learning methods in distinguishing HRGB and non-HRGB behaviors, with AUROC as the evaluation metric. Factor Significance Evaluation: The importance of active and passive features is assessed using an ablation method. For significant factors, AME is applied to estimate their effect on HRGB severity, and PAF is used to evaluate the proportion of HRGB cases that could be prevented by intervening on these factors. Abbreviations:A, active data; AME, average marginal effects; AUROC, area under the receiver operating characteristic curve; A&P, active and passive data; DT, Decision Tree; HRGB, high-risk gaming behaviors; LR, Logistic Regression; NB, Naïve Bayes; P, passive data; PAF, population attributable fraction; RF, Random Forest; SVM, Support Vector Machine.

Recently, there has been growing interest in leveraging digital technologies to enhance mental health monitoring and diagnostics. Wearable devices provide a critical window into autonomic and behavioral patterns that are typically altered in mental health conditions by passively collecting continuous, multidimensional data [10]. Changes in sleep continuity and reduced physical activity have been consistently linked to depressive disorders [11,12], while elevated resting heart rate variability has been associated with anxiety [13]. However, most research to date has focused on traditional mental health disorders, such as depression and anxiety [14], with less attention paid to behavioral addictions, particularly HRGB [15]. This gap is noteworthy given the global rise in gaming engagement and its associated harms. This study aimed to bridge this gap by examining whether wearable data can improve the identification of university students with HRGB. We aimed to benchmark multimodal (self-report + wearable) predictions against traditional methods (only self-report) using machine learning to identify key wearable-derived risk factors and explore their preventive utility. An overview of this study is presented in Fig. 1. This approach integrates digital phenotyping with predictive analytics, contributing to digital psychiatry and paving the way for early and tailored interventions.

## 2. Methods and Materials

### 2.1 Participants and Recruitment

Participants were recruited simultaneously between September 2023 and February 2024 through advertisements placed at a university in Shanghai and a local university in Zhuzhou, Hunan Province. The participants were divided into two groups: HRGB and non-HRGB. The inclusion criteria for both groups were as follows: (1) current enrollment as a university student; (2) no restriction on gender; (3) voluntary participation in HRGB screening, with a Gaming Disorder Screening Scale (GDSS) total score ≥30 for the HRGB group or <25 for the non-HRGB group [16]; (4) ownership of an Android smartphone, agreement to wear a Huawei smartwatch (Huawei Watch GT3 or Wristband6, Huawei Technologies Co., Ltd., Shenzhen, Guangdong, China) with physiological monitoring functions, and consent to download the research app and upload data; (5) agreement to participate in the study with written informed consent provided. GDSS is an 18-item instrument with a total score range of 18–72 that was developed and psychometrically validated specifically for screening gaming disorder in Chinese adolescents and young adults. It has demonstrated robust reliability and validity in clinical assessments. Based on previous findings from our research team, individuals with GDSS scores in the top 20% (≥30) were classified as the HRGB group, while those with scores below the mean (<25) were classified as the non-HRGB group [17]. Participants who scored between 25 and 29 were excluded from the study. The exclusion criteria for both groups were as follows: (1) history of head trauma with loss of consciousness, epilepsy, or other severe or unstable medical conditions; (2) past or current diagnosis of severe psychiatric disorders such as schizophrenia, mood disorders, intellectual disability, and others; (3) previous use of psychoactive substances, such as cocaine and methamphetamine, excluding tobacco and alcohol, or the use of other dopaminergic medications. Baseline data were collected in three domains: (1) demographic characteristics (gender and age); (2) gaming behaviors (gaming days per week, daily gaming duration and gaming expenditure); (3) clinical scales, including GDSS, Patient Health Questionnaire-9 (PHQ-9) to assess depressive symptoms, Generalized Anxiety Disorder-7 (GAD-7) to measure anxiety symptoms, and Pittsburgh Sleep Quality Index (PSQI) to evaluate sleep quality.

### 2.2 Time-Based Ecological Momentary Assessment (t-EMA)

Participants were instructed to log in daily to complete the t-EMA within a designated window (18:00–23:00), which took approximately 2 min. PHQ-9 and GAD-7 scales were administered on days 7, 14, 21, and 28. PHQ-9 is a self-report instrument used to screen for major depressive disorder and assess the severity of depressive symptoms. GAD-7 is a brief self-administered questionnaire designed to screen for generalized anxiety disorder and measure the severity of anxiety symptoms. These two scales were used to assess the general emotional state of the participants during the study period and represent important dimensions to evaluate gaming addiction behaviors. To ensure compliance, the completion status was monitored, and reminders were sent for missed submissions. A tiered incentive scheme was used: weekly bonuses ( $ 0.70–1.40) were awarded for full compliance, and a final bonus ( $ 11–16) was awarded for completing all 28 days, with the exact amounts based on overall compliance and data quality. Technical details regarding the development of the t-EMA application are described in **Supplementary Methods**.

### 2.3 Monitoring Metrics

In this study, physiological and psychological data were collected using validated active and passive measurement tools, resulting in 24 continuous variables. To enhance the performance of machine learning classification, we draw on methods from previous studies [18,19,20]. All variables were aggregated over time into mean and variance features. Active assessments included PHQ-9 for depression and GAD-7 for anxiety symptoms. Passive data were collected using a smartwatch or wristband. The study utilized daily average values obtained from Huawei’s health platform, encompassing the following metrics: blood oxygen (peripheral capillary oxygen saturation (SpO_2_): max/min/mean), sleep architecture (N1/N2, N3, and rapid eye movement (REM) percentages), physical activity (daily calories and steps), and a heart rate index (max/min ratio) [21,22]. Crucially, all data represent daily averages, and no modeling of rhythmic, trend-based, or change-point temporal structures was performed. The complete list of variables is presented in **Supplementary Table 1**.

### 2.4 Data Preprocessing

Following a rigorous recruitment and data filtering process, 60 participants were enrolled in the study, yielding 15,738 valid temporal data points. Details on participant attrition and data filtering are described in the **Supplementary Methods** section. The MissForest algorithm was used to handle missing values while preserving as many of the original data characteristics as possible. This non-parametric, machine-learning-based imputation method is suitable for mixed-type data (continuous and categorical) [23]. After imputation, descriptive statistics and distributional comparisons were conducted to assess the consistency between the original and imputed datasets.

### 2.5 Statistical Analysis

#### 2.5.1 Difference Comparison

Group differences in the 24 metrics between non-HRGB and HRGB groups were assessed using Independent Samples *t*-tests (for normally distributed data) or Mann-Whitney U tests (for non-parametric data). To account for multiple comparisons, *p*-values were false discovery rate (FDR)-corrected using the Benjamini–Hochberg procedure, with adjusted *p*-values < 0.05 deemed significant. All analyses were conducted in R (version 4.2.0; R Foundation for Statistical Computing, Vienna, Austria).

#### 2.5.2 Machine Learning Classification Task

Binary classification prediction was performed using Python (version 3.12.3; Python Software Foundation, Wilmington, DE, USA) with the Scikit-learn library (version 1.5.2; scikit-learn developers, Inria, Saclay, France) to distinguish between the participant groups. Accordingly, three distinct classification trials were conducted for each binary classification task using only actively collected data as input features, only passively collected data, and the full combined dataset. Multiple machine learning models were tested, including Logistic Regression (LR), Support Vector Machine (SVM), Decision Tree (DT), Random Forest (RF), Naïve Bayes (NB), and XGBoost. The data were randomly split into independent training and test sets with stratification (1:1 ratio), and standardization was performed during training to avoid data leakage. The model training and hyperparameter tuning were performed using nested cross-validation. The inner layer employed repeated stratified five-fold cross-validation (with three repeats) for GridSearchCV from scikit-learn to select the optimal parameters using the area under the receiver operating characteristic curve (AUROC) as the metric, and the outer layer conducted the final evaluation on the independent test set. To further quantify performance stability, 1000 bootstrap resamplings were applied to the test set predictions, and the 95% confidence interval (CI) for AUROC was calculated. All analyses were performed using Python along with libraries, including Scikit-learn and XGBoost (version 2.1.0; XGBoost Contributors, University of Washington, Seattle, WA, USA). A fixed random seed was set throughout the study to ensure the reproducibility of the results.

#### 2.5.3 Importance Prediction

In this study, an ablation method was used to assess feature importance by randomly permuting each feature’s values across subjects and breaking its association with the target, such as a psychiatric diagnosis. The resulting decrease in model performance was measured using AUROC, with a larger decrease indicating greater importance. The importance score for each variable was defined as the average percentage decrease in the AUROC across the six machine learning models after randomization.

#### 2.5.4 Average Marginal Effects (AME)

AME was calculated to assess the impact of various indicators on the risk of gaming behavior. AME is the sample mean of the partial derivatives of the regression equation with respect to a given explanatory variable. For continuous outcomes, such as the GDSS score, AME corresponds to the change per one-standard-deviation increase in the predictor. Predicted outcomes for the target sleep feature were displayed in adjusted prediction plots, with other covariates held at their median values and varied by ±2 standard deviations. All models were estimated using a Bayesian framework with Stan (version 2.33.0; Stan Development Team, Columbia University, New York, NY, USA), accessed via the R package brms (version 2.22.0; Paul-Christian Bürkner, University of Stuttgart, Stuttgart, Germany) [24]. All models were estimated using 40,000 sampling iterations, with thinning applied by retaining every 10th draw. Weakly informative priors centered at zero were employed to constrain the estimates within plausible ranges and to provide statistical regularization by shrinking coefficients toward zero [25]. The posterior samples of AME were summarized using an 89% credible interval (CrI). All available outcome data were retained under the assumption of missing data at random. To evaluate the potential impact of gender on the results, a sensitivity analysis was performed using data from males only, while controlling for gender as a covariate.

#### 2.5.5 Population Attributable Fraction (PAF)

To assess the potential population-level preventive impact of modifying the indicators, the adjusted PAF was calculated as the proportion of incident HRGB cases that could be prevented if a specific exposure were eliminated. Each continuous feature was dichotomized by classifying the upper quartile as exposed (1) and the lower three quartiles as unexposed (0), yielding binary variables, such as “high” versus “low depressive symptoms”. A confounder-adjusted binary LR model was fitted using the glm function in R, and the adjusted PAF was computed using AFglm function from the R package AF (version 0.1.5; Karolinska Institutet, Stockholm, Sweden). Additionally, a sensitivity analysis was conducted using data from male participants only, with gender included as a covariate, to assess its potential influence on the outcomes.

## 3. Results

**Fig. 2.**
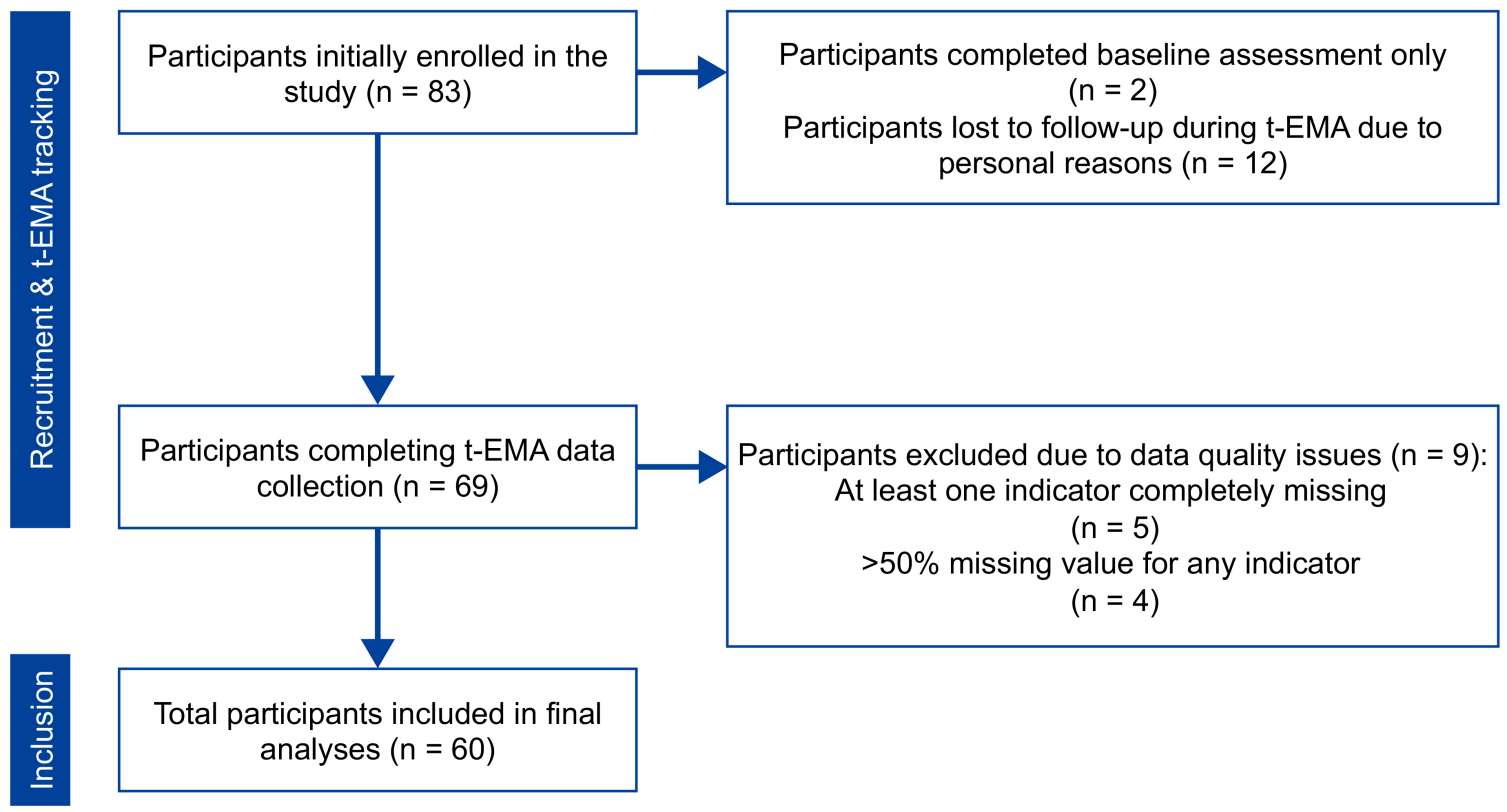
**Flowchart of participant recruitment, t-EMA tracking, and inclusion in the study**. A total of 83 participants were recruited and assigned to either the HRGB (n = 52) or non-HRGB group (n = 31). For the HRGB group, 2 participants completed baseline assessments only, while 50 continued to the t-EMA phase. Of these, 43 completed t-EMA data collections, and 37 provided complete data for all indicators, resulting in 37 participants included in the final analysis. For the non-HRGB group, all 31 participants continued to the t-EMA phase, with 26 completing t-EMA data collection and 23 providing complete data for all indicators, resulting in 23 participants included in the final analysis. Abbreviations: t-EMA, time-based Ecological Momentary Assessment.

Initially, 83 undergraduate students were enrolled in this study. After rigorous exclusion procedures (Fig. 2 and **Supplementary Methods**), a final sample of 60 participants with complete and valid data was retained, yielding 15,738 valid dynamic data points.

Overall, this study included 23 participants in the non-HRGB group and 37 in the HRGB group. Significant differences were observed between the two groups in terms of demographic characteristics (gender), gaming behaviors (daily gaming time), and clinical scale scores (GDSS, PHQ-9, GAD-7, and PSQI). The details are presented in Table 1.

**Table 1.** **Demographic and baseline characteristics of the study participants**.

			Control group (n = 23)	High risk group (n = 37)	χ^2^ or T value	*p* value
Demographic characteristics	Gender	Male	12 (52.17%)	34 (91.89%)	χ^2^ (1) = 12.50	<0.00
		Female	11 (47.83%)	3 (8.11%)		
	Age		21.09 ± 3.03	18.43 ± 1.34	4.66	<0.00
Gaming behaviors	Game days per week		3.74 ± 2.83	6.16 ± 1.48	4.35	<0.00
	Game time per day		1.28 ± 1.22	4.26 ± 2.32	5.67	<0.00
	Game spending		29.78 ± 52.75	65.11 ± 85.08	1.79	0.08
Clinical scales	GDSS		20.78 ± 2.50	36.92 ± 4.69	15.17	<0.00
	PHQ-9		2.57 ± 3.11	5.16 ± 3.91	2.70	0.01
	GAD-7		1.44 ± 2.29	3.65 ± 3.28	2.83	0.01
	PSQI		11.00 ± 3.78	15.32 ± 6.19	3.02	<0.01

Abbreviations: GDSS, Gaming Disorder Screening Scale; PHQ-9, Patient Health Questionnaire-9; GAD-7, Generalized Anxiety Disorder-7; PSQI, Pittsburgh Sleep Quality Index.

### 3.1 Differences Between HRGB and Non-HRGB Groups on Active and Passive Indicators

**Fig. 3.**
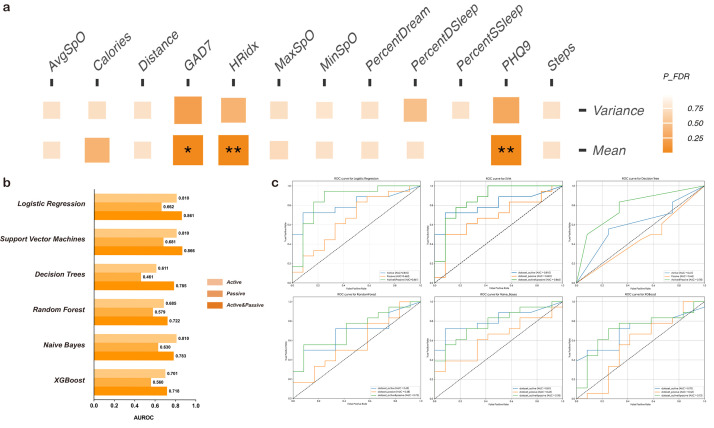
**Group comparisons of active and passive indicators and machine learning classification of HRGB**. (a) Comparative analysis of 24 active and passive indicators between the HRGB and non-HRGB groups. Square size and color depth correspond to *p*_FDR values (smaller values → larger squares and darker colors). (b) Performance comparison of machine learning models for classifying HRGB versus non-HRGB groups under different predictor conditions (active indicators only, passive indicators only, and combined). (c) ROC curves for six machine learning models in predicting HRGB: Logistic Regression, SVM, Decision Trees, Random Forest, Naïve Bayes, and XGBoost. Each panel displays model performance using active indicators only (blue), passive indicators only (orange), and combined indicators (green). In panel (a), * indicates *p*_FDR < 0.05, and ** indicates *p*_FDR < 0.01. Abbreviations: AvgSpO, average SpO_2_; SpO_2_, peripheral capillary oxygen saturation; HRidx, heart rate index; MaxSpO, maximum SpO_2_; MinSpO, minimum SpO_2_; PercentDream, percentage of REM sleep in total sleep time; PercentDSleep, percentage of deep sleep (N3 stage of Non-Rapid Eye Movement Sleep, NREM) in total sleep time; PercentSSleep, percentage of shallow sleep (N1/N2 stages of NREM) in total sleep time; ROC, receiver operating characteristic.

Multiple active (self-reported) and passive (wearable-sensor-derived) indicators were compared between HRGB and non-HRGB groups to characterize group-specific profiles. In terms of active indicators assessed via psychological questionnaires, the HRGB group exhibited significantly higher mean scores on the PHQ-9 (5.00 versus 1.79; *p*_FDR = 0.01) and GAD-7 (3.37 versus 1.12; *p*_FDR = 0.02) than the non-HRGB group (Fig. 3a), indicating a greater burden of psychological distress in the HRGB group. Additionally, the mean heart rate index (HRidx_mean) was significantly elevated in the HRGB group (2.03 versus 1.68; *p*_FDR = 0.01; Fig. 3a), further supporting heightened physiological or psychological stress. Although differences in blood oxygen (SpO_2_: max/min/mean), sleep architecture (N1/N2, N3, and REM percentages), and physical activity (daily calories and steps) were observed, they were not statistically significant after FDR correction. Details of the differences between non-HRGB and HRGB groups are provided in **Supplementary Table 2**.

### 3.2 Passive Indicators Enhance Machine Learning Performance in Classifying HRGB Group

We used multiple machine learning approaches to investigate the utility of passive indicators for identifying individuals with HRGB. Specifically, we framed the task as a binary classification problem to distinguish between the HRGB and non-HRGB groups. We used six algorithms to build the predictive models: LR, SVM, DT, RF, NB, and XGBoost. Three sets of predictor variables were evaluated: (1) four active indicators only, (2) 20 passive indicators only, and (3) all 24 combined indicators (**Supplementary Materials**).

The results indicated that combining active and passive indicators yielded the best predictive performance in five of the six models (LR, SVM, DT, RF, and XGBoost), outperforming the models using either data type alone (Fig. 3b,c; **Supplementary Tables 3–10**). The AUROC for LR increased to 0.86 (combined) from 0.81 (active) and 0.67 (passive), a trend that was mirrored by the SVM, DT, and RF.

For the XGBoost model, the combined data approach yielded only a marginally higher performance (by 0.02) than using active data alone. However, for the NB model, the combined performance was slightly lower than the active-only model. These findings underscore that integrating passive sensing data with self-reports generally enhances the accuracy of identifying HRGB.

### 3.3 Heart Rate, Sleep, and Depressive Symptoms as Key Predictors

**Fig. 4.**
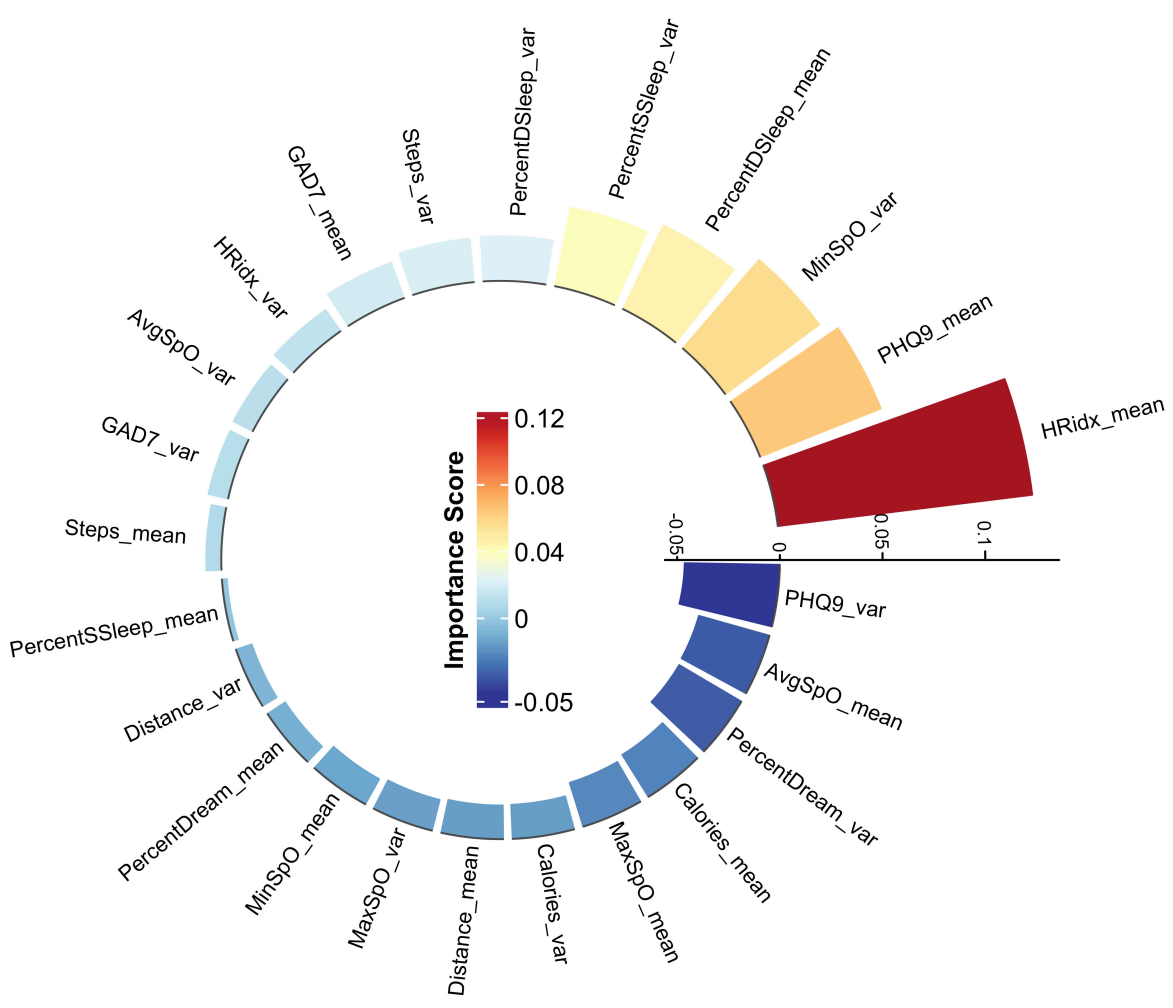
**Feature importance ranking derived from ablation analysis across machine learning models. ** The importance of each variable is quantified by the mean decrease in AUROC following permutation. Full abbreviations are provided in **Supplementary Material**.

We performed an ablation analysis to quantify the relative importance of each feature, identify the most influential predictors and enhance model interpretability. The results revealed that the top five most important predictors were the mean heart rate index (HRidx_mean; mean AUROC decrease, 0.12; 95% CI, 0.00 to 0.25), mean PHQ-9 score (PHQ9_mean, 0.06; 95% CI, −0.05 to 0.18), variance of minimum SpO_2_ (MinSpO_var, 0.06; 95% CI, −0.06 to 0.17), mean percentage of deep sleep (PercentDSleep_mean, 0.05; 95% CI, −0.07 to 0.16), and variance of percentage of light sleep (PercentSSleep_var, 0.04; 95% CI, −0.11 to 0.19; Fig. 4; **Supplementary Table 11**). These findings corroborate prior evidence highlighting the relevance of psychological and physiological markers in predicting HRGB and provide empirical support for refining predictive models to improve their accuracy and reliability.

### 3.4 Protective Effects of Improved Mood, Sleep, and Heart Rate Regulation on Gaming Behavior

We calculated the AME of each variable on GDSS scores to quantify the magnitude of the association between key predictors and gaming severity. Higher mean PHQ-9 scores were positively associated with GDSS scores: a one-standard-deviation increase in the PHQ-9 score was associated with an average increase of 3.508 in the GDSS score (89% CrI [1.85, 5.20]). Similarly, a higher mean heart rate index was associated with increased GDSS scores (AME = 1.36; 89% CrI [0.13, 2.60]; **Supplementary Table 12**). The findings were not significantly influenced by gender (**Supplementary Tables 13,14** for gender-related analyses).

**Fig. 5.**
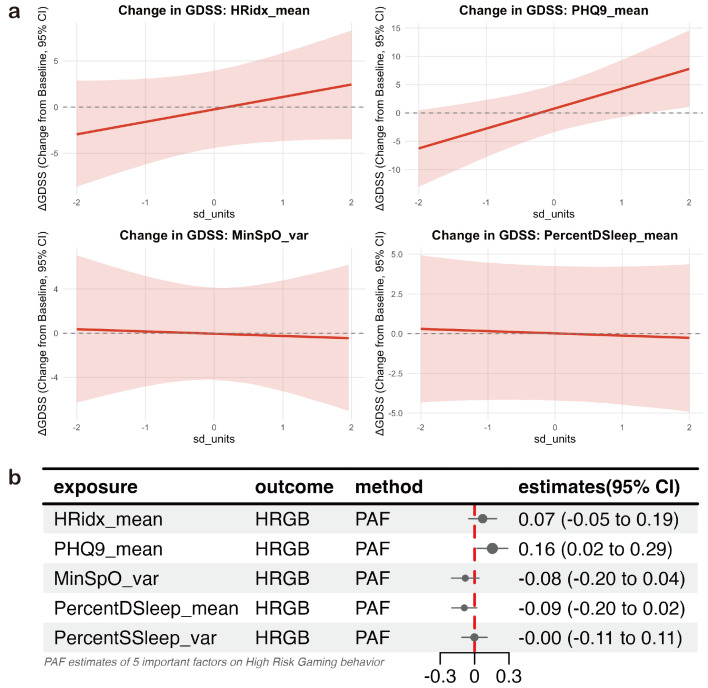
**Adjusted associations with GDSS scores and PAFs for modifiable risk factors of HRGB**. (a) Adjusted prediction plots for GDSS scores across a two-standard-deviation range of key predictors, with all other covariates held at their median values. Effect of mean heart rate index (HRidx_mean), mean PHQ-9 score (PHQ9_mean), variance of minimum oxygen saturation (MinSpO_var), and mean percentage of deep sleep (PercentDSleep_mean). (b) Forest plot of PAF and corresponding 95% confidence intervals for modifiable risk factors associated with HRGB. Abbreviations: CrI, credible interval; CI, confidence interval; PercentSSleep_var: Variance of Percentage of Light Sleep.

The adjusted prediction plots (Fig. 5) illustrate the modeled relationships between the selected active and passive features and GDSS scores, with all other covariates held at their median values. Above-average PHQ-9 scores and heart rate index were associated with higher GDSS scores, whereas higher proportions of deep sleep and greater variance in minimum SpO_2_ were associated with lower GDSS scores. The effect of the variance in the light sleep percentage on the GDSS scores was relatively small.

Furthermore, we estimated the potential public health impact of interventions targeting these modifiable factors (**Supplementary Table 15**). For the PHQ-9 mean, PAF was 15.51% (95% CI, 2.04%–28.99%), indicating that reducing depressive symptoms could prevent a significant proportion of HRGB at the population level. In contrast, the 95% CIs for PAFs of the other four predictors included zero, suggesting insufficient evidence for a significant preventive effect through intervention on these parameters. The findings were not significantly influenced by gender (**Supplementary Tables 16,17** for gender related analysis).

## 4. Discussion

This study innovatively used passively collected data from wearable wristbands to track and predict HRGB patterns among young adults. Six machine learning models were employed to compare three data modalities (active, passive, and combined) for classifying individuals as HRGB versus non-HRGB. After hyperparameter tuning, models using only active or passive data exhibited moderate performance (AUROC). In contrast, integrating both data types improved the predictive accuracy: the combined approach outperformed the active-only models in five of the six classifiers. The superior performance of multimodal digital phenotyping stems from the advantages of wearable-based data acquisition, which provides continuous, objective measurements less affected by recall and social desirability biases. Data collection using wearables is convenient, less biased, objective, highly comparable, and easily scalable and replicable, facilitating cross-study validation and comparison [26]. Moreover, physiologically grounded metrics from wearables may help patients understand the biological basis of their diagnosis and the connection between behavior and symptoms. In this study, the classification of HRGB and non-HRGB groups was based on an extreme-group approach using within-sample GDSS scores to enhance phenotypic contrast for machine-learning classification. Previous studies have also demonstrated that HRGB individuals identified by this method significantly differ from controls in areas such as time perception and perceived social support. However, the intermediate score range of 25–29 may still contain feature information relevant to HRGB individuals. Future studies should explore the relationship between continuous GDSS scores and individual digital phenotypes in larger samples to facilitate precise prevention strategies.

These findings identified the level of depressive symptoms as a significant predictor in machine learning models, differentiating the HRGB group from the non-HRGB group. The AME analysis revealed that higher depressive symptoms, indicated by elevated PHQ-9 scores, were significantly associated with increased GDSS scores, consistent with the existing literature. A longitudinal follow-up study identified depressive symptoms as independent predictors of gaming disorder incidence [27]. Furthermore, at the individual level, childhood depressive symptoms have been demonstrated to positively predict subsequent gaming disorder symptoms [28]. Consequently, early identification, assessment, and intervention targeting depressive symptoms may be critical strategies for preventing the onset of gaming disorders. This study further quantified the potential preventive effect of reducing depression levels using PAF analysis. The results estimated that effectively lowering the depressive symptoms in the HRGB group to the level of the non-HRGB group, via evidence-based interventions such as cognitive behavioral therapy [7], prevented approximately 15.5% (95% CI, 2.04%–28.99%) of individuals from developing HRGB. These findings substantiate the therapeutic potential of targeting depressive symptoms as a complementary strategy for mitigating HRGB, highlighting the centrality of emotional regulation in gaming disorder etiology and supporting integrated treatment approaches.

The heart rate index was also identified as important for the machine-learning classification task. Within two standard deviations, an increase in the heart rate index was associated with an increase in the estimated GDSS score, indicating a positive correlation between the heart rate index and HRGB. Based on the available device data, we defined the heart rate index as the ratio of the maximum to minimum heart rate within a day. Previous research has linked sympathetic nervous system activity to this index, which reflects a broader range of autonomic nervous fluctuations. Inhibiting sympathetic activity (using beta-blockers) in elderly hypertensive patients reduced the 24-h heart rate amplitude and the max/min ratio, whereas enhancing sympathetic tone (using calcium channel blockers) significantly increased the heart rate amplitude and raised this ratio [29]. Studies in animal models and simulations have similarly demonstrated that suppressing sympathetic activity reduces the daily heart rate amplitude and max/min ratio in mice [30,31]. Multiple studies have found increased sympathetic activity and vagal inhibition in individuals with gaming or other psychiatric disorders [22,32]. Accordingly, the higher max/min heart rate ratio observed in the HRGB group in this study may be associated with elevated sympathetic excitability. These findings suggest a potential link between the HRGB and autonomic nervous system dysregulation. However, they fail to clarify whether the widespread autonomic changes are a direct manifestation of HRGB or a compensatory response. This distinction is critical, as the concepts of dynamic biological interactions and compensatory pathways are highly relevant across neurological and behavioral disorders. Given evidence of such compensatory mechanisms in other contexts [33], future studies should specifically investigate whether the autonomic changes observed in HRGB represent a direct effect or a compensatory response. Elucidating this distinction is crucial, as it could facilitate the identification of more specific digital biomarkers for HRGB.

Sleep has been consistently associated with mood, stress, and Internet gaming disorders. Adolescents and young adults with gaming disorders tend to have higher PSQI scores (indicating poorer sleep quality) [4]. Neuroimaging evidence revealed that sleep influences gaming behavior via disrupted hippocampal connectivity, particularly with the nucleus accumbens and prefrontal-cortical regions, implicating memory, reward, and cognitive control pathways [34]. Poor sleep quality is a recognized risk factor for gaming disorders [35]. This study further revealed that sleep disturbances manifest concurrently with the emergence of HRGB in young adults. At baseline, affected individuals demonstrated significantly higher PSQI scores, while subsequent machine-learning analyses identified a reduced proportion of deep sleep (N3 stage of Non-Rapid Eye Movement Sleep) as a significant predictor of HRGB. These findings position sleep architecture as a viable biomarker for early risk identification. Wearable devices offer a practical means of detecting these physiological signatures during the initial stage. Future investigations should examine the dynamic evolution of sleep parameters as the progression from HRGB to gaming disorder and evaluate the preventive efficacy of sleep-focused interventions in mitigating the development of pathological gaming.

### Limitations

This study has several methodological and ethical limitations that warrant careful consideration. The limited sample size and significant gender imbalance, such as male predominance in the HRGB group, may compromise both the representativeness of the identified predictive features and model generalizability, particularly in female populations. Future studies should address the limitations of sample size and gender imbalance, such as male predominance in the HRGB group, using gender-balanced sampling and conducting gender-stratified analyses, thereby enhancing the robustness of predictive models and validating the external generalizability of these findings across more diverse populations. From a data quality perspective, irregularities in device wear and occasional sensor detachment introduce non-random missingness and measurement noise. Although rigorous data-cleaning protocols were applied, residual biases may persist, potentially compromising feature robustness and model performance. Furthermore, the continuous passive monitoring of physiological and behavioral parameters raises substantive ethical questions regarding data privacy, secure storage infrastructure, the scope of informed consent, and the boundaries of commercial data application. In the absence of established regulatory frameworks, reconciling data utility with the protection of participants’ rights remains a critical prerequisite for ethically sustainable large-scale implementation.

## 5. Conclusions

This study systematically evaluated the utility of digital phenotyping in identifying HRGB by integrating passively collected physiological data from wearable devices with actively reported psychobehavioral indicators. The results have shown that machine learning models incorporating multimodal data performed best at distinguishing between HRGB and non-HRGB individuals, with depressive symptoms, heart rate index, and deep sleep proportion presenting significant predictive values. Early intervention targeting depressive symptoms may have the potential to prevent HRGB. These findings not only confirm the advantages of wearable devices in enabling continuous and objective monitoring of behavior and physiological states and provide empirical support for early screening and preventive interventions based on digital phenotyping. The feasibility of this approach is enhanced by the non-invasive nature and widespread adoption of consumer-grade wearables (e.g., smart bands) among adolescents, which facilitates large-scale monitoring in naturalistic settings and strengthens ecological validity. Future research should further validate the robustness and generalizability of these biobehavioral markers by increasing sample sizes, extending observation periods, and balancing gender distribution. Additionally, exploring their potential applications in clinical and public health contexts, such as integrating them into routine school health screenings or outpatient follow-up monitoring, could advance precision prevention and personalized interventions for gaming disorders.

## Data Availability

The datasets used and analyzed during the current study are available from the corresponding authors on reasonable request.
