# Supplementary material for: Digital Phenotyping of High-Risk Gaming Behavior Using Wearable Devices
Source: Alpha Psychiatry. 2026 Jun 30;27(3):48256. doi: 10.31083/AP48256 (PMC13339874; doi:10.31083/AP48256)
Supplement: Supplementary file 1 [file 2757-8038-27-3-48256-s1.zip › Supplementary Materials.docx]

**Supplementary Materials**

**Supplementary Methods:**

**Development of t-EMA Application**

Developed in collaboration with Huawei Technologies Co., Ltd., the system for monitoring gaming behavior and mental health consists of three components: a Huawei smart wearable device, a user-end mobile application (Game Behavior and Mental Health App), and an administrator web portal. The Huawei wearable devices (including Huawei Band 6, Band 7, or Watch GT3) were used to record participants’ daily sleep metrics (total sleep duration, deep sleep duration), physical activity (total calories burned, total steps), and heart rate data (maximum and minimum heart rates). The user-end app collected daily questionnaire responses and, upon participant authorization, retrieved health data (sleep and activity) from the wearable device. It also allowed users to view and upload wristband data. The administrator portal was used to manage participant accounts, upload and push daily questionnaires to the app, monitor data upload progress, track questionnaire completion, and download datasets.

**Data quality control**

Following a rigorous recruitment process, a total of 83 university students were initially enrolled in the study. Among them, two participants completed only the baseline questionnaire and did not continue with the subsequent phases, while 12 withdrew during the t-EMA tracking period due to personal reasons. The remaining 69 participants with complete data were included in a consolidated dataset comprising 24 descriptive indicators. After excluding individuals with any missing values on these indicators, 64 participants remained. To mitigate the impact of missing data on further analyses, we applied a conventional threshold excluding any participant with more than 50% missing values across the 28-day monitoring period for any variable, resulting in a final sample of 60 participants and 15,738 valid dynamic data points.

**Supplementary Tables:**

**Supplementary Table 1. List of Abbreviations**

| **ABB** | **Full Name** |
| --- | --- |
| PHQ9_var | Patient Health Questionnaire-9 (Variance) |
| PHQ9_mean | Patient Health Questionnaire-9 (Mean) |
| GAD7_var | Generalized Anxiety Disorder-7 (Variance) |
| GAD7_mean | Generalized Anxiety Disorder-7 (Mean) |
| AvgSpO_mean | Average Peripheral Capillary Oxygen Saturation (Mean) |
| AvgSpO_var | Average Peripheral Capillary Oxygen Saturation (Variance) |
| MaxSpO_mean | Maximum Peripheral Capillary Oxygen Saturation (Mean) |
| MaxSpO_var | Maximum Peripheral Capillary Oxygen Saturation (Variance) |
| MinSpO_mean | Minimum Peripheral Capillary Oxygen Saturation (Mean) |
| MinSpO_var | Minimum Peripheral Capillary Oxygen Saturation (Variance) |
| PercentDream_mean | Percentage of Dream Sleep (Mean) |
| PercentDream_var | Percentage of Dream Sleep (Variance) |
| PercentSSleep_mean | Percentage of Light Sleep (Mean) |
| PercentSSleep_var | Percentage of Light Sleep (Variance) |
| PercentDSleep_mean | Percentage of Deep Sleep (Mean) |
| PercentDSleep_var | Percentage of Deep Sleep (Variance) |
| HRidx_mean | Heart Rate Index (Mean) |
| HRidx_var | Heart Rate Index (Variance) |
| Calories_mean | Calories Expenditure (Mean) |
| Calories_var | Calories Expenditure (Variance) |
| Distance_mean | Distance Traveled (Mean) |
| Distance_var | Distance Traveled (Variance) |
| Steps_mean | Step Count (Mean) |
| Steps_var | Step Count (Variance) |

**Supplementary Table 2. Difference Comparison between Health Control and High Risk Gaming Behavior Group**

| **Parameters** | **Health_Group** | **High_Risk_Group** | **P_Value** | **FDR_Value** |
| --- | --- | --- | --- | --- |
| PHQ9_mean | 1.79 | 5.00 | 0.00 | 0.00 |
| GAD7_mean | 1.12 | 3.37 | 0.00 | 0.02 |
| MaxSpO_mean | 99.91 | 99.87 | 0.26 | 0.70 |
| MinSpO_mean | 89.68 | 88.91 | 0.69 | 0.75 |
| AvgSpO_mean | 97.25 | 97.10 | 0.64 | 0.75 |
| Steps_mean | 4642.31 | 4965.93 | 0.54 | 0.75 |
| Distance_mean | 3183.38 | 3411.78 | 0.54 | 0.75 |
| Calories_mean | 151.90 | 208.99 | 0.11 | 0.37 |
| PercentSSleep_mean | 0.46 | 0.46 | 0.98 | 0.98 |
| PercentDSleep_mean | 0.29 | 0.29 | 0.62 | 0.75 |
| PercentDream_mean | 0.20 | 0.21 | 0.41 | 0.75 |
| HRidx_mean | 1.68 | 2.03 | 0.00 | 0.00 |
| PHQ9_var | 2.63 | 5.93 | 0.06 | 0.29 |
| GAD7_var | 1.43 | 3.52 | 0.03 | 0.20 |
| MaxSpO_var | 0.08 | 0.13 | 0.33 | 0.73 |
| MinSpO_var | 22.40 | 17.25 | 0.74 | 0.77 |
| AvgSpO_var | 0.36 | 0.34 | 0.67 | 0.75 |
| Steps_var | 8962333.20 | 9781464.58 | 0.69 | 0.75 |
| Distance_var | 4107106.60 | 4635912.93 | 0.59 | 0.75 |
| Calories_var | 12921.90 | 34156.58 | 0.34 | 0.73 |
| PercentSSleep_var | 0.00 | 0.00 | 0.58 | 0.75 |
| PercentDSleep_var | 0.00 | 0.00 | 0.16 | 0.48 |
| PercentDream_var | 0.00 | 0.00 | 0.39 | 0.75 |
| HRidx_var | 0.17 | 0.22 | 0.09 | 0.37 |

**Supplementary Table 3. Optimal Performance and Parameters of the Logistic Regression Model**

| **Dataset** | **Accuracy** | **Precision** | **Recall** | **AUC Value** | **Optimal Parameters** |
| --- | --- | --- | --- | --- | --- |
| Active | 0.73 | 0.81 | 0.72 | 0.81 | {'C': 0.1, 'penalty': 'l2', 'solver': 'liblinear'} |
| Passive | 0.60 | 0.60 | 1.00 | 0.66 | {'C': 0.01, 'penalty': 'l2', 'solver': 'newton-cg'} |
| Active&Passive | 0.80 | 0.83 | 0.83 | 0.86 | {'C': 10, 'penalty': 'l2', 'solver': 'newton-cg'} |

**Supplementary Table 4. Optimal Performance and Parameters of the SVM Model**

| **Dataset** | **Accuracy** | **Precision** | **Recall** | **AUC Value** | **Optimal Parameters** |
| --- | --- | --- | --- | --- | --- |
| Active | 0.73 | 0.81 | 0.72 | 0.81 | {'C': 0.01, 'dual': True, 'loss': 'squared_hinge', 'penalty': 'l2'} |
| Passive | 0.63 | 0.67 | 0.78 | 0.68 | {'C': 10, 'dual': True, 'loss': 'squared_hinge', 'penalty': 'l2'} |
| Active&Passive | 0.77 | 0.76 | 0.89 | 0.87 | {'C': 1.0, 'dual': True, 'loss': 'squared_hinge', 'penalty': 'l2'} |

**Supplementary Table 5. Optimal Performance and Parameters of the Decision Trees Model**

| **Dataset** | **Accuracy** | **Precision** | **Recall** | **AUC Value** | **Optimal Parameters** |
| --- | --- | --- | --- | --- | --- |
| Active | 0.63 | 0.77 | 0.56 | 0.61 | {'max_features': 'sqrt', 'min_samples_leaf': 2, 'min_samples_split': 5, 'splitter': 'best'} |
| Passive | 0.47 | 0.56 | 0.50 | 0.46 | {'max_features': 'sqrt', 'min_samples_leaf': 1, 'min_samples_split': 7, 'splitter': 'best'} |
| Active&Passive | 0.77 | 0.79 | 0.83 | 0.78 | {'max_features': 'sqrt', 'min_samples_leaf': 5, 'min_samples_split': 2, 'splitter': 'best'} |

**Supplementary Table 6. Optimal Performance and Parameters of the Random Forest Model**

| **Dataset** | **Accuracy** | **Precision** | **Recall** | **AUC Value** | **Optimal Parameters** |
| --- | --- | --- | --- | --- | --- |
| Active | 0.63 | 0.68 | 0.72 | 0.69 | {'max_depth': 5, 'max_features': 'sqrt', 'min_samples_leaf': 1, 'min_samples_split': 2, 'n_estimators': 100} |
| Passive | 0.63 | 0.62 | 1.00 | 0.58 | {'max_depth': 7, 'max_features': 'sqrt', 'min_samples_leaf': 1, 'min_samples_split': 2, 'n_estimators': 100} |
| Active&Passive | 0.63 | 0.64 | 0.89 | 0.72 | {'max_depth': 5, 'max_features': 'sqrt', 'min_samples_leaf': 1, 'min_samples_split': 5, 'n_estimators': 100} |

**Supplementary Table 7. Optimal Performance and Parameters of the Naïve Bayes Model**

| **Dataset** | **Accuracy** | **Precision** | **Recall** | **AUC Value** | **Optimal Parameters** |
| --- | --- | --- | --- | --- | --- |
| Active | 0.73 | 0.92 | 0.61 | 0.81 | {'var_smoothing': 1.0} |
| Passive | 0.57 | 0.78 | 0.39 | 0.63 | {'var_smoothing': 0.0005336699231206307} |
| Active&Passive | 0.63 | 1.00 | 0.39 | 0.78 | {'var_smoothing': 1.0} |

**Supplementary Table 8. Optimal Performance and Parameters of the XGBoost Model**

| **Dataset** | **Accuracy** | **Precision** | **Recall** | **AUC Value** | **Optimal Parameters** |
| --- | --- | --- | --- | --- | --- |
| Active | 0.60 | 0.60 | 1.00 | 0.70 | {'colsample_bytree': 0.8, 'learning_rate': 0.01, 'max_depth': 3, 'n_estimators': 50, 'subsample': 0.8} |
| Passive | 0.60 | 0.62 | 0.89 | 0.56 | {'colsample_bytree': 1.0, 'learning_rate': 0.2, 'max_depth': 3, 'n_estimators': 200, 'subsample': 0.8} |
| Active&Passive | 0.60 | 0.60 | 1.00 | 0.72 | {'colsample_bytree': 0.8, 'learning_rate': 0.01, 'max_depth': 3, 'n_estimators': 50, 'subsample': 0.8} |

**Supplementary Table 9: Repeated Stratified K-Fold Cross-Validation AUROC Distribution**

| dataset | mdoel | AUROC_mean | AUROC_std |
| --- | --- | --- | --- |
| active | DecisionTree | 0.66 | 0.19 |
| active | LogisticRegression | 0.81 | 0.17 |
| active | NaïveBayes | 0.79 | 0.18 |
| active | RandomForest | 0.69 | 0.19 |
| active | SVM | 0.81 | 0.17 |
| active | XGBoost | 0.73 | 0.17 |
| active_passive | DecisionTree | 0.63 | 0.20 |
| active_passive | LogisticRegression | 0.68 | 0.18 |
| active_passive | NaïveBayes | 0.64 | 0.21 |
| active_passive | RandomForest | 0.76 | 0.17 |
| active_passive | SVM | 0.68 | 0.19 |
| active_passive | XGBoost | 0.77 | 0.21 |
| passive | DecisionTree | 0.52 | 0.21 |
| passive | LogisticRegression | 0.48 | 0.30 |
| passive | NaïveBayes | 0.51 | 0.24 |
| passive | RandomForest | 0.51 | 0.20 |
| passive | SVM | 0.50 | 0.28 |
| passive | XGBoost | 0.51 | 0.26 |

**Supplementary Table 10. Classification Model Performance: Test AUC, CV and Bootstrapping Results on Three Data Sources**

| **dataset** | **model** | **test_auc** | **cv_auc_mean** | **cv_auc_std** | **bootstrap_auc_mean** | **ci_lower** | **ci_upper** |
| --- | --- | --- | --- | --- | --- | --- | --- |
| **active** | **LogisticRegression** | **0.70** | **0.81** | **0.22** | **0.70** | **0.49** | **0.89** |
| **active** | **SVM** | **0.73** | **0.82** | **0.21** | **0.73** | **0.52** | **0.91** |
| **active** | **DecisionTree** | **0.59** | **0.70** | **0.25** | **0.59** | **0.39** | **0.79** |
| **active** | **RandomForest** | **0.56** | **0.66** | **0.28** | **0.56** | **0.34** | **0.77** |
| **active** | **Na**ï**veBayes** | **0.75** | **0.78** | **0.25** | **0.75** | **0.52** | **0.94** |
| **active** | **XGBoost** | **0.66** | **0.70** | **0.25** | **0.66** | **0.45** | **0.87** |
| **passive** | **LogisticRegression** | **0.49** | **0.56** | **0.26** | **0.49** | **0.28** | **0.70** |
| **passive** | **SVM** | **0.49** | **0.54** | **0.26** | **0.49** | **0.28** | **0.71** |
| **passive** | **DecisionTree** | **0.47** | **0.47** | **0.21** | **0.47** | **0.26** | **0.68** |
| **passive** | **RandomForest** | **0.41** | **0.53** | **0.27** | **0.41** | **0.20** | **0.65** |
| **passive** | **Na**ï**veBayes** | **0.58** | **0.55** | **0.23** | **0.57** | **0.37** | **0.77** |
| **passive** | **XGBoost** | **0.44** | **0.44** | **0.23** | **0.45** | **0.22** | **0.68** |
| **active_passive** | **LogisticRegression** | **0.56** | **0.68** | **0.21** | **0.56** | **0.34** | **0.76** |
| **active_passive** | **SVM** | **0.56** | **0.67** | **0.19** | **0.57** | **0.37** | **0.79** |
| **active_passive** | **DecisionTree** | **0.29** | **0.55** | **0.24** | **0.29** | **0.15** | **0.45** |
| **active_passive** | **RandomForest** | **0.61** | **0.68** | **0.22** | **0.61** | **0.38** | **0.83** |
| **active_passive** | **Na**ï**veBayes** | **0.63** | **0.66** | **0.20** | **0.64** | **0.42** | **0.83** |
| **active_passive** | **XGBoost** | **0.65** | **0.75** | **0.27** | **0.65** | **0.41** | **0.87** |

**Supplementary Table 11. Results of the Ablation Study**

| **IDX** | **Logistic Regression** | **SVM** | **Decision Tree** | **Random Forest** | **Na**ï**ve Bayes** | **XGBoost** | **ΔAVG** |
| --- | --- | --- | --- | --- | --- | --- | --- |
| PHQ9_var | -0.01 | 0.01 | 0.04 | -0.17 | -0.05 | -0.10 | -0.05 |
| AvgSpO_mean | -0.02 | -0.04 | -0.04 | -0.06 | 0.02 | -0.07 | -0.04 |
| PercentDream_var | -0.01 | -0.05 | 0.05 | -0.11 | -0.07 | -0.03 | -0.04 |
| Calories_mean | 0.00 | -0.04 | 0.00 | -0.08 | 0.02 | -0.04 | -0.02 |
| MaxSpO_mean | -0.04 | -0.03 | 0.02 | -0.15 | 0.06 | 0.00 | -0.02 |
| Calories_var | 0.01 | 0.02 | 0.09 | -0.14 | -0.01 | -0.07 | -0.02 |
| Distance_mean | 0.03 | 0.01 | 0.00 | -0.07 | 0.00 | -0.07 | -0.02 |
| MaxSpO_var | -0.04 | -0.03 | 0.00 | 0.02 | -0.01 | -0.04 | -0.02 |
| MinSpO_mean | 0.03 | -0.03 | 0.00 | -0.01 | -0.02 | -0.05 | -0.01 |
| PercentDream_mean | -0.03 | -0.03 | 0.09 | 0.00 | -0.05 | -0.05 | -0.01 |
| Distance_var | -0.02 | 0.01 | 0.04 | -0.03 | 0.05 | -0.10 | -0.01 |
| PercentSSleep_mean | 0.03 | 0.02 | 0.00 | 0.04 | -0.01 | -0.10 | 0.00 |
| Steps_mean | 0.05 | 0.07 | 0.04 | -0.06 | -0.01 | -0.05 | 0.01 |
| GAD7_var | 0.03 | 0.05 | 0.00 | -0.04 | 0.05 | -0.03 | 0.01 |
| AvgSpO_var | 0.05 | 0.06 | 0.00 | -0.04 | 0.01 | -0.01 | 0.01 |
| HRidx_var | 0.01 | 0.02 | 0.15 | -0.07 | 0.02 | -0.05 | 0.01 |
| GAD7_mean | 0.04 | 0.04 | 0.05 | -0.07 | 0.07 | -0.02 | 0.02 |
| Steps_var | 0.03 | 0.03 | 0.23 | -0.12 | -0.02 | -0.03 | 0.02 |
| PercentDSleep_var | 0.04 | 0.05 | 0.00 | 0.04 | 0.04 | -0.03 | 0.02 |
| PercentSSleep_var | 0.01 | 0.05 | 0.30 | 0.04 | -0.04 | -0.13 | 0.04 |
| PercentDSleep_mean | 0.05 | 0.07 | 0.24 | -0.06 | -0.02 | -0.01 | 0.05 |
| MinSpO_var | 0.13 | 0.12 | 0.13 | -0.06 | 0.12 | -0.11 | 0.06 |
| PHQ9_mean | 0.01 | 0.01 | 0.28 | 0.01 | 0.05 | 0.02 | 0.06 |
| HRidx_mean | 0.11 | 0.09 | 0.36 | 0.03 | 0.07 | 0.08 | 0.12 |

**Supplementary Table 12. AME Estimations**

| **Variable** | **AME_Mean_ΔGDSS** | **89%CrI_Lower** | **89%CrI_Upper** |
| --- | --- | --- | --- |
| HRidx_mean | 1.36 | 0.13 | 2.60 |
| PHQ9_mean | 3.51 | 1.85 | 5.20 |
| MinSpO_var | -0.22 | -1.88 | 1.43 |
| PercentDSleep_mean | -0.14 | -0.69 | 0.42 |
| PercentSSleep_var | 0.00 | -0.03 | 0.03 |

AME: Average Marginal Effect

**Supplementary Table 13. AME Estimations male only**

| **Variable** | **AME_Mean_ΔGDSS** | **89%CrI_Lower** | **89%CrI_Upper** |
| --- | --- | --- | --- |
| HRidx_mean | 1.12 | -0.16 | 2.40 |
| PHQ9_mean | 3.89 | 2.19 | 5.60 |
| MinSpO_var | -0.26 | -1.95 | 1.42 |
| PercentDSleep_mean | -0.17 | -0.75 | 0.43 |
| PercentSSleep_var | 0.00 | -0.03 | 0.03 |

**Supplementary Table 14. AME Estimations gender as covariate**

| **Variable** | **AME_Mean_ΔGDSS** | **89%CrI_Lower** | **89%CrI_Upper** |
| --- | --- | --- | --- |
| HRidx_mean | 1.36 | 0.12 | 2.62 |
| PHQ9_mean | 3.53 | 1.85 | 5.19 |
| MinSpO_var | -0.21 | -1.87 | 1.45 |
| PercentDSleep_mean | -0.14 | -0.69 | 0.41 |
| PercentSSleep_var | 0.00 | -0.03 | 0.03 |

**Supplementary Table 15. PAF Results**

| **exposure** | **outcome** | **method** | **estimates** | **L_95%CI** | **U_95%CI** |
| --- | --- | --- | --- | --- | --- |
| HRidx_mean | HRGB | PAF | 0.07 | -0.05 | 0.19 |
| PHQ9_mean | HRGB | PAF | 0.16 | 0.02 | 0.29 |
| MinSpO_var | HRGB | PAF | -0.08 | -0.20 | 0.04 |
| PercentDSleep_mean | HRGB | PAF | -0.09 | -0.20 | 0.02 |
| PercentSSleep_var | HRGB | PAF | 0.00 | -0.11 | 0.11 |

PAF: Population Attributable Fraction; HRGB: High Risk Gaming Behavior

**Supplementary Table 16. PAF Results (male only)**

| **exposure** | **outcome** | **method** | **estimates** | **L_95%CI** | **U_95%CI** |
| --- | --- | --- | --- | --- | --- |
| HRidx_mean | HRGB | PAF | 0.06 | -0.05 | 0.16 |
| PHQ9_mean | HRGB | PAF | 0.14 | 0.03 | 0.24 |
| MinSpO_var | HRGB | PAF | -0.05 | -0.15 | 0.05 |
| PercentDSleep_mean | HRGB | PAF | -0.04 | -0.15 | 0.06 |
| PercentSSleep_var | HRGB | PAF | -0.01 | -0.10 | 0.09 |

**Supplementary Table 17. PAF Results (gender as covariate)**

| **exposure** | **outcome** | **method** | **estimates** | **L_95%CI** | **U_95%CI** |
| --- | --- | --- | --- | --- | --- |
| HRidx_mean | HRGB | PAF | 0.05 | -0.06 | 0.15 |
| PHQ9_mean | HRGB | PAF | 0.16 | 0.03 | 0.29 |
| MinSpO_var | HRGB | PAF | -0.06 | -0.16 | 0.05 |
| PercentDSleep_mean | HRGB | PAF | -0.07 | -0.16 | 0.02 |
| PercentSSleep_var | HRGB | PAF | 0.02 | -0.09 | 0.13 |
